# Emergence of perceptuomotor relationships during paleolithic stone toolmaking learning: intersections of observation and practice

**DOI:** 10.1038/s42003-021-02768-w

**Published:** 2021-11-11

**Authors:** Kristel Yu Tiamco Bayani, Nikhilesh Natraj, Nada Khresdish, Justin Pargeter, Dietrich Stout, Lewis A. Wheaton

**Affiliations:** 1 School of Biological Sciences, Georgia Institute of Technology, Atlanta, GA USA; 2 Division of Neurology, UCSF Weill Institute for Neurosciences, San Francisco, CA USA; 3 Anthropology Department, Emory University, Atlanta, GA USA; 4 Department of Anthropology, New York University, New York, NY USA

**Keywords:** Object vision, Motor control

## Abstract

Stone toolmaking is a human motor skill which provides the earliest archeological evidence motor skill and social learning. Intentionally shaping a stone into a functional tool relies on the interaction of action observation and practice to support motor skill acquisition. The emergence of adaptive and efficient visuomotor processes during motor learning of such a novel motor skill requiring complex semantic understanding, like stone toolmaking, is not understood. Through the examination of eye movements and motor skill, the current study sought to evaluate the changes and relationship in perceptuomotor processes during motor learning and performance over 90 h of training. Participants’ gaze and motor performance were assessed before, during and following training. Gaze patterns reveal a transition from initially high gaze variability during initial observation to lower gaze variability after training. Perceptual changes were strongly associated with motor performance improvements suggesting a coupling of perceptual and motor processes during motor learning.

## Abstract (web summary)

In order to evaluate changes and relationships in perceptual process during motor learning and motor performance over training, Bayani et al examine eye movements and motor skill in a paleolithic stone toolmaking task. They demonstrate the importance of understanding the emergence and evolution of perceptual-motor coupling not only in complex motor skill learning.

## Introduction

Linnaeus chose to name our species “wise,” but it may be the ability to learn from others that makes Homo sapiens unique^1^. Humans live in a cultural niche of artificial landscapes, structures, artifacts, skills, practices, and beliefs constructed over generations and far exceeding the understanding or creative potential of any individual. Paleolithic stone tools provide our earliest evidence of this emerging niche and the social learning of increasingly complex skills and knowledge that supports it^2–4^. Acquisition of such demanding technical skills requires the generative interaction of observation and practice^5^. Still, relatively little is known about the interaction of observation and practice generally^6^ or for stone tool making specifically^7,8^. To address this, we used eye-tracking to investigate gaze patterns during stone tool making action observation. Our sample comprises 11 research participants with no prior stone tool making experience learning to make Paleolithic stone “handaxes”^3^ comparable to those made by Homo heidelbergensis ca. 500,000 years ago^9,10^. Pargeter et al. (2019, 2020) have published details of training and learning outcomes elsewhere. Here we address variability in gaze both at the group level over time and in relation to individual technological success and motor performance metrics.

Motor variability is an important part of exploratory behavior^6,11,12^ that supports individual skill acquisition^13,14^. Assessment of motor variability has figured prominently in attempts to understand the interaction of individual and social learning in the intergenerational reproduction of stone tool making techniques^5,15,16^. Researchers know much less about perceptual variability, exploration, and learning of stone tool making^17^, thus the contribution of demonstration and observation to social transmission remains unclear^2,16,18^.

Stone tool making through controlled fracture (“knapping”) requires the sequential detachment of flakes from a stone core using precise strikes with a handheld hammer (typically stone, bone, or antler). Each removal leaves traces that archeologists can use to reconstruct discrete actions and sequential strategies. These strategies range in complexity from simple iteration to multi-level goal hierarchies needed to produce later Paleolithic forms^17^, such as the stone “handaxes” studied here. Executing such strategies requires reliable control over individual flake removals, including the sensitive adaptation of kinematics to variable core morphology, composition, and positioning^7,15^. Therefore, learning to knap requires mastery of subtle interactions between bodily kinematics, object properties, action outcomes, and technological goals^7,17,19^. These factors might be facilitated by observation of expert performance^5^ and reflected in the spatiotemporal variation of gaze patterns.

Attentional processes drive eye movements that facilitate foveation on salient and meaningful visual features^20,21^, thus providing a window on cognitive processes^22^ that we exploit in order to better understand processes of observational learning. Work on the role of gaze in motor control and learning has largely focused on action planning and execution^12,22,23^ or goal inference (e.g.^24^), At least one recent study^25^ has reported gaze variation’s effects on observational learning of a novel task (prosthesis use). Bayani et al found that eye movements tracking action path and bodily effector rather than the action start and endpoints during demonstration was associated with more efficient kinematics during execution. This provides support for a role in gaze in maximizing observational learning through motor resonance. Arbib (2011) has advanced similar proposals for the role of observation in knapping skill acquisition. It remains unknown to what extent useful information about the rapid and finely tuned kinematics and stone fracture mechanics^7,15^ underlying knapping skill can be extracted by observing others^5^.

To investigate this issue, we evaluated changes in (1) gaze variability and (2) the relationship between gaze patterns and knapping performance over training. Participants (*n* = 11) received up to 90 h of practice and direct active teaching^26^ from an expert knapper. At three time points (Pre = before training, Post 1 = 50 h training, Post 2 = after completion), we invited participants to observe a 17-min video of their instructor producing a handaxe while we recorded participant gaze behavior. A small number of prior studies have examined gaze variability during motor learning, although not of entirely novel tasks like knapping. These studies find that the standard deviation of raw eye velocity demonstrated a general reduction in gaze variability with training^27^. Gaze entropy, a value that utilizes the standard deviation and fixation distribution, provided an additional measure of exploratory behavior^28^. We thus hypothesized that if participants were extracting useful technical information from knapping observation, we should observe: 1) a reduction in gaze variability with training and 2) greater spatial allocation towards more meaningful features of the task. We further predicted stronger associations between perceptual processes and motor performance with practice.

## Results

### Determination of meaningful action sequences

**Fig. 1:**
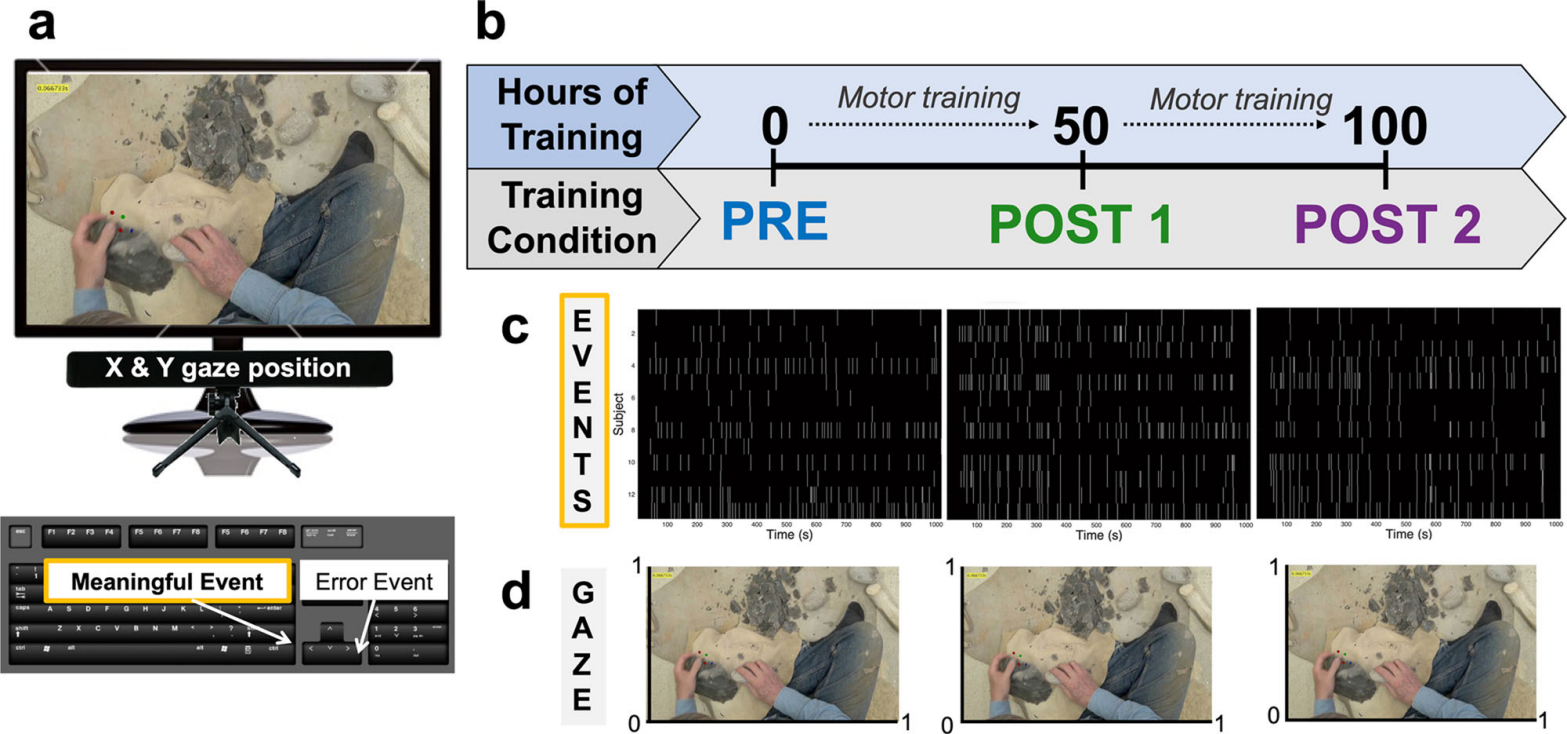
Experimental design and task. **a** Action observation was presented on a computer monitor with the Gazepoint eye tracker and keyboard situated directly in front of the participants. The image shows a single, sample frame of the video. Their task was to indicate an meaningful and error event using the left and right arrow on the keyboard respectively. **b** The training paradigm involved 100 h of training with participants viewing the video and performing the task. The Pre condition is the baseline training condition with no hours of training, Post 1 occurred after 50 h of training, and Post 2 occurred after 100 h of training. **c** One dimension of data displays meaningful key presses during the video observation across all three training conditions are displayed to display time points in the video that participants perceive to be meaningful. **d** Gaze data is expressed in two dimensional (x,y) coordinates in the 0,1 dimension.

**Fig. 2:**
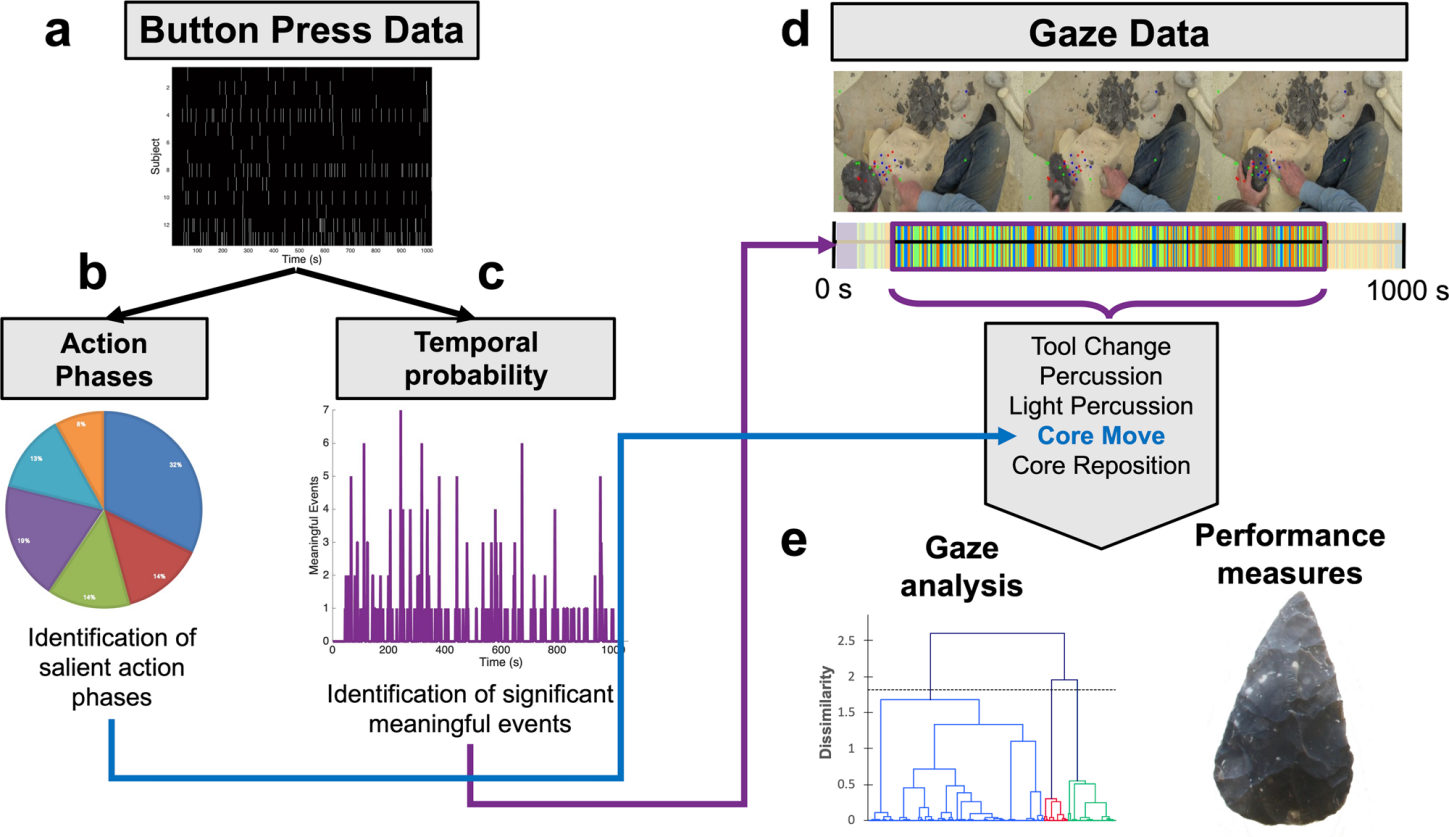
Data preprocessing pipeline. **a** Participants’ response to “meaningful events” via keyboard button press was used to reduce the data and isolate sections of gaze data based salient time points and action phases. **b** Button press data corresponded to one of six different types of action phases. This was used to quantify which action phase was more salient to participants (see Fig. 2 for action phase saliency). Specific time points in the video were also isolated based on the frequency of button presses within a given time point. **c** A sample histogram using Post 2 button press data is shown with time on the x-axis and frequency of button presses on the y-axis. Clear peaks represent time points with the highest participant response. **d** Action phases and time points from button press data was used to pinpoint specific action phases and time points in gaze data for **e** gaze analysis. Performance measures, specifically model score, were assessed during time points around their respective assessments.

Participants watched a 17-min video of an expert knapper performing the stone toolmaking task before, during, and after training. The same expert flintknapper that trained all participants (NK) categorized each second in the video. This allowed for the temporal categorization of action phases based on one of the six action phases: core reposition, core move, light percussion, percussion, grinding, and tool change (see Methods). During video observation, we instructed participants to press a button whenever they judged the knapper to have “completed a meaningful unit of action in pursuit of [the] goal” of making a handaxe (Fig. 1a). We matched these button presses to specific time points and motor actions produced by the actor (Fig. 1c, Fig. 2). This allowed us to identify action phases important to the participants used for our analysis without bias from the investigators. Meaningful button presses across all participants and training conditions revealed a high degree of responses corresponding to core move time points.

**Fig. 3:**
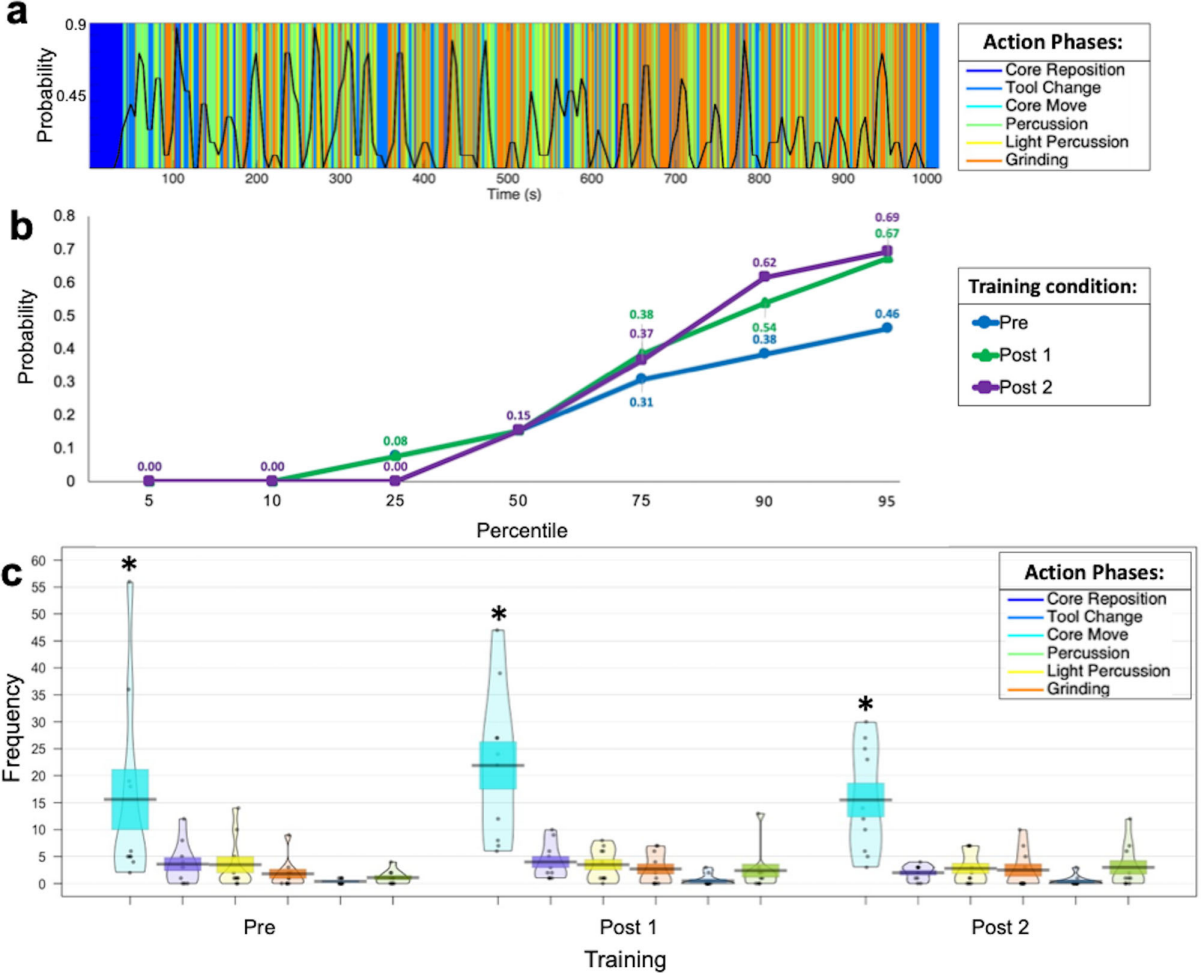
Data-driven approach to identify salient action phases and time points. **a** Each second of the video was categorized as one of the six action phases. Each color represents a single action phase over time. The black line represents the probability of meaningful events calculated using a 10-s window with a 5-s slide. **b** We isolated core move time points with high probability in a 10-s window. **c** The mean number (±S.E.) of counts across subjects for each action phase. Individual dots represent data points and the shaded area is a kernel density estimate of the distribution. Figure reveals core moves are the most salient action phase that most participants responded to regardless of training condition.

The study used participants’ reactions to meaningful time points in the video to generate a group-level temporal probability plot by isolating a 10-s window with a 5-s overlap. Within each window, we calculated the probability by dividing the number of participants that indicated a particular time segment contained a meaningful event and dividing it by the total number of participants (Fig. 3a, black trace). We determined time points for more in-depth analysis by assigning the 90th percentile as the threshold for meaningful time points across all participants (Fig. 3b). The meaningful time points were also matched to specific action phases with greater meaningful button presses associated with core moves (χ2(5) = 131.24, *p* < 0.01, Fig. 3c) compared to core reposition (t(5) = 9.31, *p* < 0.001, g = 1.22), light percussion (t(5) = 9.33, *p* < 0.001, g = 1.17), grinding (t(5) = 10.03, *p* < 0.001, g = 1.50), tool change (t(5) = 11.46, *p* < 0.001, g = 1.65), and percussion (t(5) = 10.23, *p* < 0.001, g = 1.44). Together, core move time points that met the 90th percentile probability threshold or higher were isolated and extracted for continuous gaze analysis (Fig. 2, Fig. 3b, c).

### Gaze variability

We sought to understand any patterns of dispersion of gaze data across a given core move time point to address gaze variability. Temporal dispersion of gaze enables us to identify how the continuous gaze data vary over training. Spatial dispersion will determine where gaze falls during observation.

### Temporal dispersion of gaze

We evaluated group-level changes in gaze patterns with the highest probability of meaningful button presses by implementing a hierarchical clustering algorithm of gaze positions over core move time points and calculating cluster variance for each meaningful core move time point. Implementation of hierarchical clustering (see Methods) across time points revealed changes in variability of gaze within a cluster (within-class variance) and variability of gaze between different clusters (between-class variance) across training. While within-class gaze variance decreased from Pre to Post 1 and increased from Post 1 to Post 2, a one-way ANOVA did not reach statistical threshold across any of the training phases. Dissimilarity demonstrated no main effect of training but displayed a stepwise reduction as participants progressed through training.

**Fig. 4:**
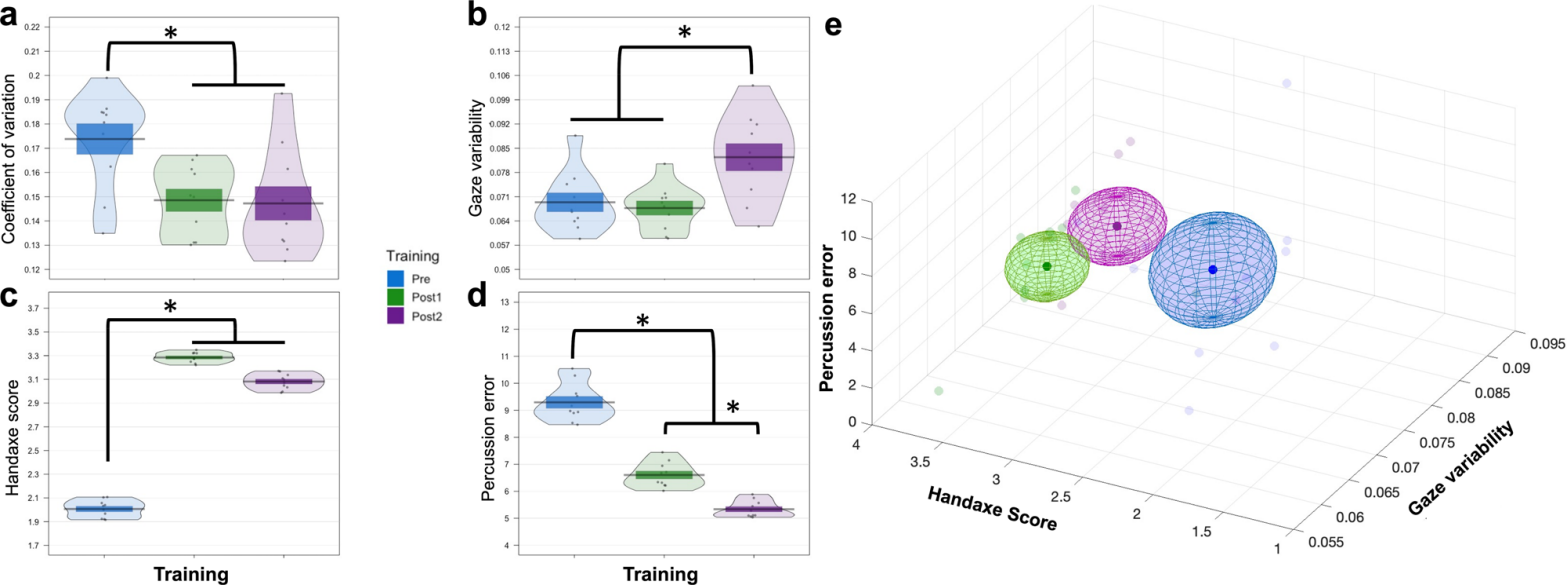
Perceptual-motor changes after 0 (Pre), 50 (Post 1), and 100 (Post 2) hours of training. Bar plots represent mean ± S.E. Individual dots represent data points and the shaded area is a kernel density estimate of the distribution. **a** Gaze coefficient of variation (mean ± S.E.) across an entire core move time point decreased with training while **b** gaze variability (mean ± S.E.) relative to the core preparation site increased in Post 2. **c** Participants ability to create a stone tool, or model score (mean ± S.E.), increased after 50 h of training, and **d** their ability to predict action outcomes (mean ± S.E.) improved due to a decrease in percussion score in a step-wise fashion. **e** Outcomes from these perceptual-motor parameters reveal a perceptual-motor curve with a 3-dimensional average (model score, percussion score, gaze variability) in blue for Pre, green for Post 1, and purple for Post 2. The shaded region around the average represents the 3-dimensional 95% confidence interval (model score, percussion score, gaze variability) in blue for Pre, green for Post 1, and purple for Post 2.

These non-significant changes with training may also reflect the substantial participant-level differences in learning documented by Pargeter et al (2019,2020), which likely contributed to the large variability seen in group-level data. We thus measured gaze coefficient of variation to capture the variability of gaze data for each participants’ continuous gaze data for core move time points across training. One-way ANOVA revealed a main effect of Training (F_2,30_ = 3.74, *p* < 0.036, ƞ2 = 0.20). Pairwise comparison using TukeyHSD indicated gaze variability decreased from Pre compared to Post 1 (*p* < 0.01, g = 0.87) and Post 2 (*p* < 0.01, g = 0.949, Fig. 4a). This reduction indicates that participants focused on a smaller area of the visual scene during core moves due to training.

### Spatial dispersion of gaze

Eye movements involve top-down processes that direct gaze to information-rich and meaningful areas in the visual scene^20,21^. It is vital to assess whether the gaze changes are associated with task-specific features in the visual scene by understanding the spatial aspects of this shifting variability over training. We sought to understand the spatial dispersion of gaze by determining if participants directed foveation towards the most relevant part of the visual scene, the working edge (i.e., a portion of the tool from which toolmakers remove flakes). We extracted the working edge’s continuous location using a MATLAB-based computer vision machine learning algorithm, random sampling consensus (RANSAC). This algorithm allowed us to detect the core’s x-axis edge in each image frame (“Computer Vision ToolboxTM User’s Guide R2020a” 2004^29,30^. The output from the hierarchical cluster of gaze data identified cluster centroids, a data-driven representation of the most statistically distinct gaze position and their associated time points within a core move for all participants (see Methods). We calculated gaze distance relative from the working edge by subtracting the working edge coordinates from each participants’ gaze centroid coordinate at each frame. Results show a decrease in gaze distance from the working edge. However, this decrease across training did not reach statistical significance (F_2,27_ = 0.73, *p* = 0.49). The reduced coefficient of variation along with the proclivity for the gaze to fall closer to the working edge may reveal that training encourages gaze to fall in information-rich and meaningful areas of the visual scene^20,31^. We attributed the lack of statistical significance to the high degree of variability, which prompted more in-depth spatial analysis.

We evaluated the variability of gaze along the working edge by calculating the gaze distance from the working edge across all clusters’ time centroids. The standard deviation of those distances was extracted and used as a measure of gaze variability relative to the working edge. Quantification of gaze variability using this metric not only reduced the amount of noise inherent in gaze data but provides a statistical backing for utilizing specific time points within a single core move time point. Our results revealed a main effect of Training (**χ**^2^(2) = 8.74, *p* = 0.013, ε2 = 0.21, Fig. 4a) with significantly higher gaze variability relative to the working edge in Post 2 compared to Pre (V = 49, *p* = 0.03, g = −1.00) and Post 1 (V = 53, *p* < 0.01, g = −0.93). Training thus led to an increase in gaze variability relative to the core, especially comparing Post 1 and Post 2 gaze variability.

### Performance measures

Motor skill performance over training was quantified using two primary measures: handaxe score and percussion error. The handaxe score is a multivariate ensemble measure (higher is better) of tool quality for handaxes produced during periodic skill evaluations throughout training^3^. Percussion error measures the difference between knappers’ intended and actual impact points (i.e., lower is better) during a controlled flake production task included in the skill assessments^7^.

For handaxe score, there was a main effect of Training (**χ**^2^(2) = 622.36, *p* < 2.2e-16, **∈**2 = 0.89). Pairwise comparisons using Wilcoxon signed-rank test revealed significantly lower handaxe scores in Post 1 vs Pre (V = 0, *p* < 2.2e-16, g = −19.60) and Post 2 vs Pre (V = 820, *p* < 2.2e-16, g = −14.97) (Fig. 4a). For percussion error, a Kruskal-Wallis rank sum test revealed a main effect of Training (**χ**^2^(2) = 25.81, *p* < 0.001, **∈**2 = 0.91). Post hoc comparisons using Wilcoxon signed rank test demonstrated a statistically significant decrease from Pre to Post 1 (*p* < 0.01, g = 3.87), from Post 1 to Post 2 (*p* < 0.01, g = 3.11). Post 2 was also statistically lower than Pre (*p* < 0.01, g = 5.87, Fig. 4a). In agreement with previous analyses^3,7^, both measures reveal an improvement in general skill level with training, following a classic “power curve”^32^ of rapid initial progress followed by asymptotic leveling off as learners reach local performance optima. We quantified participants’ learning rates as the slope of a linear regression of all a participant’s handaxe scores against practice hours’ square root. Because training caused participants to converge on similar performance levels after 30 h, variation in learning rate is largely driven by initial aptitude with better initial performance resulting in a flatter slope.

### Relationship between gaze patterns and knapping performance

While the above results suggest changes in gaze patterns and motor performance driven by training, further analysis seeks to understand the extent to which perceptual (gaze) and motor performance changes may be related to each other through training.

### Perceptual-motor shifts with learning

**Fig. 5:**
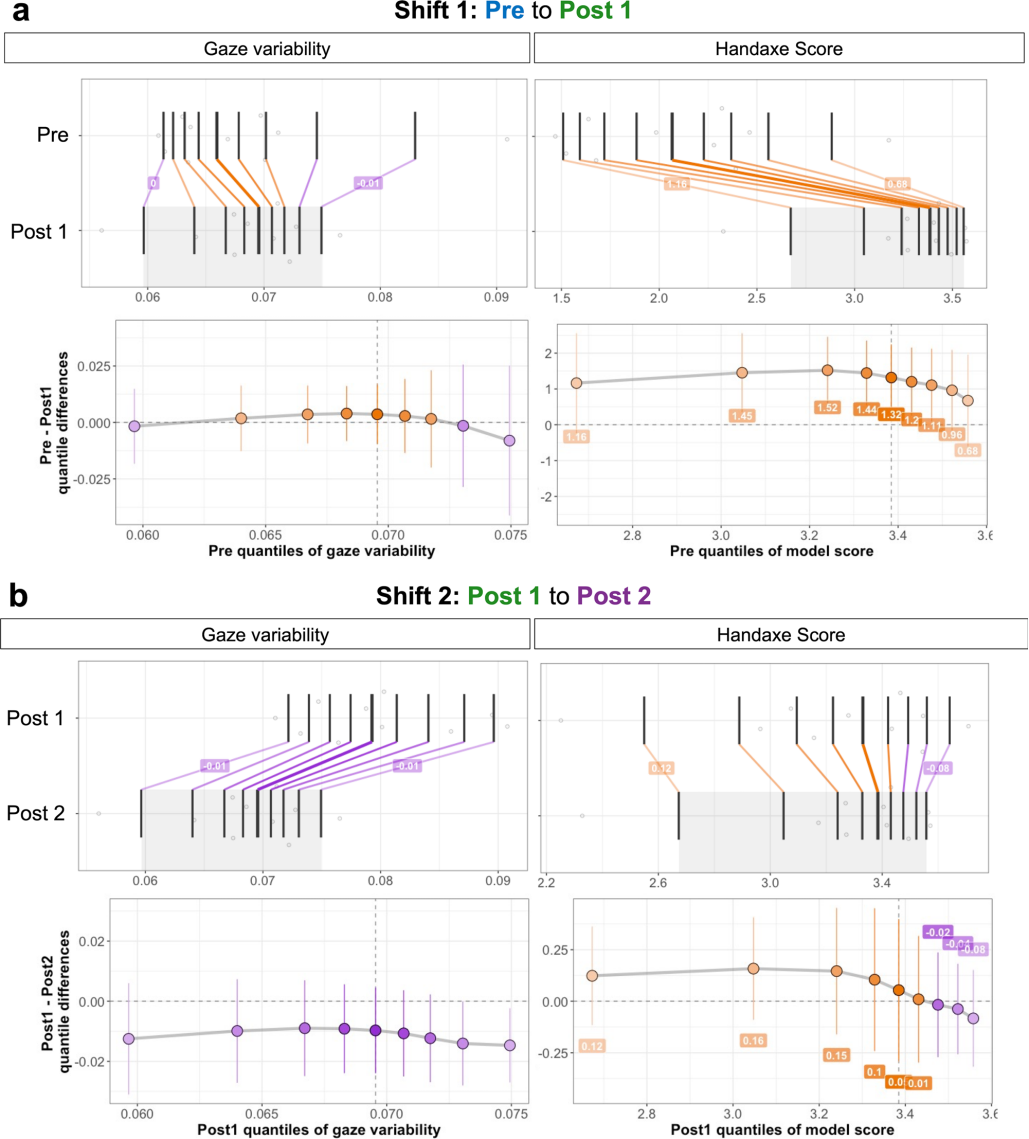
Shift function of perceptuo-motor variables. Quantification of the perceptual-motor curve along these three parameters were separated into shifts to quantify how the these parameter change from **a** Pre to Post 1 (shift 1) and from **b** Post 1 to Post 2 (shift 2) using Shift Functions using the R rogme package. In top subpanels, transparent gray dots are individual participant data points with the black lines representing decile calculated by the Harrell-Davis quantile estimator. The colored lines represent the color-coded differences between the quantiles (orange: positive, purple: negative). The bottom subpanel represents the quantile differences plotted across all quantiles between the two groups in Shift1 (Pre vs. Post1) and Shift2 (Post1 vs. Post2). The error bars represent 95% confidence interval of decile differences quantified by using percentile bootstrap.

We observed that gaze variability and handaxe quality showed a complex relationship, revealing change in motor performance followed by gaze variability changes through training (Fig. 4b–d). These visualizations prompted additional analyses to quantify the changes through shift functions, a technique implemented in neuroscience to quantify how two distributions differ^33^. We described the data using deciles wherein each condition can be separated into nine quantiles leading to the segmentation of data into ten different segments (see Methods). We divided the data into two shift matrices. Shift1 is the perceptual and performance changes from Pre to Post 1 (Fig. 4b, c), while Shift2 defines the perceptual and performance changes from Post 1 to Post 2 (Fig. 4b, d). This method allows us to quantify which specific variables undergo the most changes from one training stage to another and if the effect is applicable across all of the data. The top panel in Fig. 4c, d represents the deciles (dark black lines) in each training condition, with colored lines connecting the two conditions’ deciles representing the direction and magnitude by which that specific decile has to shift. We plotted the quantile differences to create a shift function with 0 y-axis values representing no shift (or no difference between training within a specific decile), positive (orange) representing a shift to higher values, and negative (purple) representing a shift to lower values. The error bars represent a 95% confidence interval percentile bootstrap. Handaxe score demonstrated the strongest, positive shift during the Shift1 (quantile differences: 1.16, 1.45, 1.52, 1.44, 1.32, 1.20, 1.11, 0.96, 0.68), while gaze variability relative to the working edge did not exhibit a strong shift (Fig. 5b). However, gaze variability relative to the working edge showed greater shifts in Shift2 (quantile differences: −0.013, −0.0099, −0.0090, −0.0092, −0.0097, −0.011, −0.012, −0.014, −0.015), while handaxe score was relatively consistent. This staggered relationship between changes in execution (handaxe score) and observation (gaze variability) lends support to a cyclical model of skill acquisition^34,35^. In this model, observation of expert models guides the consolidation of motor skills through individual practice, which produces enhanced perception and understanding of demonstrated actions, leading to further learning.

### Perceptual-motor canonical correlation analysis

**Fig. 6:**
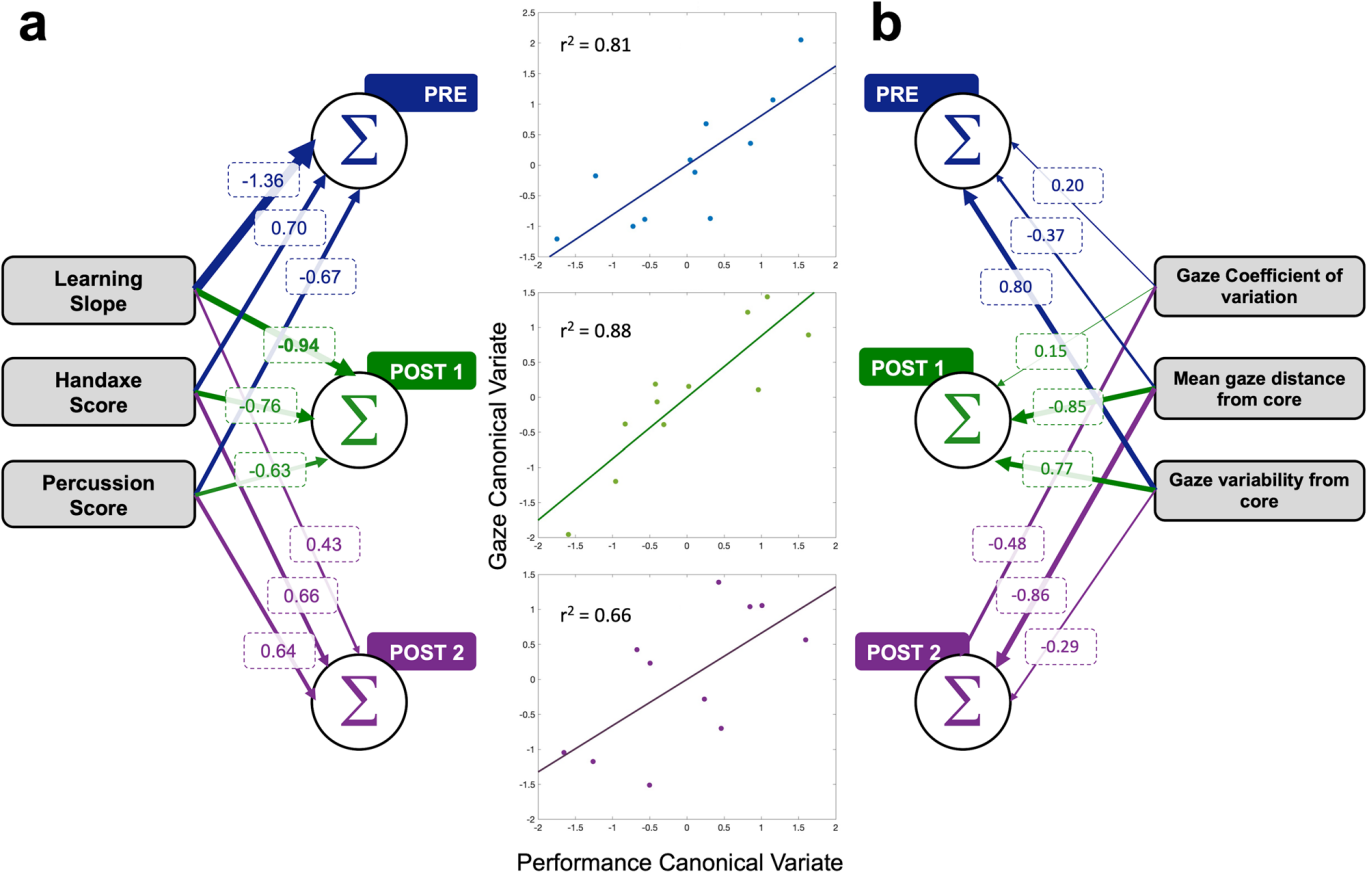
Canonical correlation analyses of perceptual and motor variables. **a** Shows the canonical weights for all performance variables, and **b** shows the canonical weights for the gaze variables. The thickness of the lines show the relative weight each variable contributes canonical cross correlation. The numbers in each box is the value of the canonical weights.

To gain a deeper understanding of the perceptual and motor relationships with training, we utilized canonical correlation analyses (CCA) to quantify the strength of the relationship between gaze and performance outcomes within a single training condition, how the relationship between variables evolved with training using Pearson’s R, and the relative contribution of each variable using canonical weights (Fig. 6). The gaze variate consisted of gaze coefficient of variation, mean distance from the core, and gaze variability from the core. The performance variate consisted of learning slope, handaxe score, and percussion error. In the Pre condition, the relationship between motor performance and gaze variables exhibited two modes that demonstrate a strong (*r*^2^ = 0.81) and moderate relationship (*r*^2^ = 0.44). The variables contributing the greatest weights in the first mode were learning slope (**ω** = −1.3762) and gaze variability from the core (**ω** = 0.7972). The relative weights of the remaining performance and gaze variables were relatively low compared to the variables mentioned above. However, the second mode demonstrated greater weights between the handaxe score (**ω** = −0.8678) and the gaze coefficient of variation (**ω** = 0.1535).

Similarly, the Post1 condition demonstrated a strong canonical correlation between the performance and gaze variables (*r*^2^ = 0.87). However, there was a single dominant mode that explained the relationship between these variables. While the highest contribution to this relationship was learning slope (**ω** = −0.9380) and mean gaze distance from core (**ω** = 0.8499), the relative contribution of handaxe score (**ω** = 0.7048), percussion error (**ω** = −0.6665), and gaze variability relative to the core (**ω** = 0.7665) was comparable. Gaze coefficient of variation had the lowest weight contributing to the correlation (**ω** = 0.1457). In the Post1 condition, we did not find a single dominant variable in each set. Instead, most of the variables in both the gaze and performance variates contributed equally to the correlation. In the Post2 condition, the canonical correlation between the gaze and performance variate decreased (*r*^2^ = 0.6618). The highest contributing variables were handaxe score (**ω** = 0.6567) and percussion error (**ω** = 0.6353) as well as mean gaze distance from core (**ω** = 0.8571).

## Discussion

Researchers have proposed that observational learning of body movement^18^ is a uniquely human capacity enabling our species’ accumulation of increasingly complex cultural and technological practices over time^36^. This relationship is likely true of ritual and communicative gestures that depend on accurate replication of arbitrary body movements^37^. There is debate on whether the learning of technical skills is more reliant on the mastery of physical affordances through individual practice^18^ and how efficiently observed movements translate effectively to different bodies^38–40^ and contexts^41,42^. This debate raises questions about the role of action observation in modern skill learning^43^ and the importance of putative neurocognitive specializations for action observation in human evolution^5^. To investigate the interaction of observation and practice in real-world technological learning, we evaluated the development of gaze patterns and motor performance during the acquisition of the completely novel, evolutionarily relevant stone tool-making skill.

Consistent with expectations, we found that training resulted in rapid initial improvements in tool making action execution accompanied by a reduction in gaze coefficient of variation (CV) during action observation. This CV reduction marks putative emergence of “quiet eye” behavior (fewer, longer fixations with fewer saccades) often associated with the development of perceptual-motor expertise^21,31,44,45^. More specifically, this eye movement pattern indicates a possible transition from exploration (sampling the visual scene) to exploitation (foveation of specific regions of the visual scene) for more in-depth processing^22,28,46^ and complex mental representation^44^. Learning and expertise are associated with the transition of attention towards exploitation due to the predictive value learned through exposure^47^. The fact that experience with tool making action execution rapidly leads participants to focus on particular aspects of observed tool making indicates a transition to the exploitation of (perceived to be) relevant technical information. It supports a potential role for action observation in learning about stone tool making object affordances and/or behavioral techniques^5^.

To further explore this potential, we examined variability in the spatial allocation of gaze over training. In contrast to decreasing eye movement variability related to the emergence of quiet eye behavior, the spatial allocation of gaze relative to the core edge became more variable over training. Furthermore, this increase in spatial variability occurred after significant performance increases observed from Pre to Post1 timepoints instead of coinciding with the Post1-Post2 period of more modest performance changes. This pattern is contrary to our predictions of decreasing variability and tighter correlation with performance derived from static visual scene studies^28^ and simple motor tasks^27^. Instead, it reflects a more complicated gaze-performance relationship characteristic of real-world skills.

Complex skills like stone tool making often involve simultaneously tracking multiple moving areas of interest (e.g., the hammerstone, anticipated point of percussion on the core edge, and core surface topography behind the edge). They also require multifocal strategies that decouple attentional mechanisms from gaze behavior^6,48,49^. Humans achieve such “visuomotor parsimony”^48^ through increased reliance on peripheral vision that allows for reduced foveation of the targets. In turn, this strategy relies on the development of detailed sensorimotor mappings sufficient to enable the extraction of information from non-foveated targets^50^. For this reason, visuomotor parsimony has been associated with late-stage skill acquisition and refinement^12^, as also seen in our study. More broadly, the complex relationships between training, gaze behavior, and performance evident in our study lend support to a cyclical model of real-world skill acquisition^34,35^. In Whiten’s (2015) model, observation of expert models guides consolidation of motor skills through individual practice^51^ leading to enhanced perception and understanding of demonstrated actions^52^. Enhanced action-perception then supports further observational learning, continuing the cycle^5^.

Finally, the canonical correlation analysis allowed us to test putative relationships between action observation and execution in individuals. Consistent with our group-level results, this demonstrated strong gaze–performance correlations during early exploration and skill acquisition (Pre and Post1) that deteriorated during later stage refinement and consolidation (Post2). Before training, a relationship between tool making aptitude coming into the study (high initial performance reflected in a relatively flat learning slope) and gaze spatial variability dominated the primary canonical mode. Thus, higher aptitude individuals anticipate to some degree the visuomotor parsimony that developed in the group as a whole over training. This anticipation suggests pre-existing sensorimotor competence in some participants. The presence of sensorimotor knowledge of a specialized skill has roots in the mirror neuron system (MNS), a frontoparietal neural network that is implicated in learning through execution and observation^53^. For example, classical ballet dancers or athletes show MNS activation during observation of their respective crafts while naïve participants failed to show MNS activation^53–55^. Furthermore, participants with prior knowledge of the task showed greater goal-directed and anticipatory gaze towards the goal of the task while naïve participants failed to show these perceptual patterns^24^. These studies provide neural and perceptual evidence for the role of observation in skill learning and expertise. Indeed, we subsequently found that initial performance correlated with self-reported years of prior experience in gross-motor crafts like carpentry and sculpture (*n* = 17, *r*^2^ = 0.39, *p* = 0.007) among all starting participants reported by Pargeter et al. (2019, 2020)^3,7^. A second pre-training canonical mode was dominated by a negative relationship between handaxe score and gaze coefficient of variation, again anticipating later group-level decreases in gaze CV associated with rising performance. Two canonical modes in the pretraining condition suggest complex influences of pre-existing sensorimotor competencies and perceptual strategies on initial stone tool making aptitude.

After 50 h of training, variation in participant performance converged at a higher level, and a single canonical mode emerged, showing a strong correlation between motor performance and gaze. This mode differs from the primary pre-training mode, most notably in the increased weight assigned to mean gaze distance from the core edge. The effect suggests that particularly successful participants adopted stable fixation points offset some distance from the moving core edge, much as expert jugglers employ a stable “gaze through” strategy fixated at a central location rather than tracking individual balls^48^. The post-training condition showed more evenly weighted performance and gaze variables, but the canonical correlation weakened from strong to moderate. This loosening of gaze-performance coupling suggests that other factors, such as differences in practice habits^3^ or the timing transitions between learning plateaus^56^, may be increasingly important in driving observed performance variation. More generally, this pattern again speaks to the complex interplay of action observation and individual practice in acquiring real-world skills^5^.

Results show that persons learning the highly skilled stone tool knapping process rapidly learn to perceive and focus on technologically informative aspects of observed tool making. Furthermore, gaze behavior during observation is strongly associated with actual tool making performance. Our study’s complex, asynchronous development of eye movement strategies, gaze spatial allocation, and toolmaking performance is consistent with a cyclical model of real-world skill acquisition. This finding has implications for debates over the role of individual vs. social learning in early stone toolmaking, and thus for interpreting the archeological evidence used to support evolutionary accounts of the origins of human culture^1,4,8,18^. It highlights the need for more real-world skill learning studies to understand perceptual-motor interactions during human skill learning.

## Methods

### Subjects

Seventeen experimental participants (10 female/7 male; 21–48 years of age, median 27.25) were recruited from Emory University (students and staff) and the surrounding community with no prior flint knapping experience. Full participation in the study amounted to ~90 h; of which, ~80 h involved training in handaxe production. Six participants left the study before the sfinal assessment with the remaining 11 achieving between 74 and 89 h training (median = 84 h). All participants were right-handed, had no prior knapping experience, and provided written informed consent. The study was approved by Emory University’s Internal Review Board (IRB study no: 00067237).

### Experimental set up

#### Workspace

Subjects were seated in front of a raised 24” computer monitor with a computer keyboard situated directly in front of the subject (Fig. 1A). Since the eye tracker is sensitive to direct sunlight and may alter pupil detection reliability, subjects faced away from the windows to avoid direct sunlight on their face and pupils. The Gazepoint control system (GP3), a 60-Hz high-performance eye tracking platform, was placed below the computer monitor approximately 65 cm or an arms distance away from the subject. The eye tracker was stabilized with a mini tripod that allowed researchers to adjust the position of the eye tracker based on each individual subjects’ height. A laptop was used to power and obtain data from the eye tracker through two USB cables.

#### Eye tracking

To ensure subjects’ distance from the computer monitor and eye tracker was consistent across all sessions and optimal for gaze data acquisition, the eye tracker is equipped with distance monitor and a 5-point calibration test. The distance monitor tracked subjects’ pupil distance from the eye tracker. A moving dot on the Gazepoint control system displayed the participants’ distance using a red or green dot indicator. A red dot indicator informs researchers if the participant was too far or too close. A green dot indicates when the participant is an optimal distance (~65 cm away) from the eye tracker. Researchers adjusted subjects’ seated position to ensure that the distance monitor’s green dot was always situated green prior to data collection. The 5-point calibration test was administered after optimal distance was determined. Subjects were instructed to follow a white dot that traveled across the computer monitor with their gaze while keeping their head completely still. Following the calibration period, five calibration markers appeared on the screen. Subjects were instructed to make saccades to 5 calibration points. Successful calibration was confirmed if gaze position fell within the bullseye of each of the 5 points. The combination of the distance monitor and 5-point calibration ensured an accuracy between 0.5 to 1 degree of the visual angle.

#### Action observation

The paradigm was executed using a Windows laptop containing the Gazepoint control and analysis system software as well as MATLAB to coordinate video replay, gaze data collection, and behavioral responses from the subjects. During all sessions, subjects viewed the same 17 min and 4 s video of an expert stone knapper creating a tear drop shaped hand axe in an egocentric viewing perspective. The video was presented using a 1680 ×1050 viewing dimension. During video observation subjects were instructed to “press the left button on the keyboard for events that seem natural and meaningful to you.” MATLAB was initiated prior to the start of the video and continued processing during video observation in order to record the timing of subjects’ meaningful and error event button presses.

### Experimental design

The behavioral and eye-tracking testing sessions were divided into three different sessions: Pre, Post 1, and Post 2 (Fig. 1b). The Pre condition is the baseline condition wherein subjects had no prior to training on the task. Their first exposure to the task was the aforementioned 17 min and 4 s video of an expert stone knapper performing the task in an egocentric perspective. Following the first session, subjects completed stone knapping training by an expert stone knapper and returned for the second session after 50 h of training for the Post 1 assessment. During this Post 1 condition, subjects observed the same video and indicated their perception of meaningful and error events with a keyboard button press. The last 50 h (100 h total) of motor training was completed before subjects came back for their third and last assessment, Post 2. During this time, subjects observed the same video and indicated their perception of meaningful and error events during video observation.

### Task

In between each training condition, subjects were trained and assessed on the flint knapping task. The goal of the task is to create a tear-dropped shaped hand-axe from a larger stone, which we will refer to as the “core” for the rest of the manuscript. All participants were instructed in basic knapping techniques including how to select appropriate percussors, initiate flaking on a nodule, maintain the correct flaking gestures and angles, prepare flake platforms, visualize outcomes, deal with raw material imperfections, and correct mistakes. Handaxe-specific instruction included establishment and maintenance of a bifacial plane, cross-sectional thinning, and overall shaping. The importance of producing thin, symmetrical pieces with centered edges was emphasized throughout the training as these are key components in successful handaxe making.

### Data preprocessing

#### Determination of meaningful action phases

While watching the videos, during Pre, Post 1 and Post 2, participants indicated their perception of meaningful actions using the left and right arrows on the computer keyboard. Keyboard button presses for meaningful events were used to pinpoint the action phases and time points that were deemed most salient to subjects. Button press latencies are time points that correspond to specific time points in the video and were collected from the start until the end of the video. Latencies were stored matrix wherein columns represented each subject and each row corresponds to a specific time the subject responded to a meaningful event. All meaningful event button press latencies were preprocessed using MALAB2015b. Latencies were rounded to the nearest second and transferred into a discrete subject x time matrix with 1 representing a button press and 0 representing no response. More specifically, a one was placed in a column (i.e., time point) within a specific row (i.e., subject) where the button press occurred. Figure 1c shows the visualization of the subject x time matrix of button press across the duration of the video for each subject (y axis) across the entire video duration (x axis) in Pre, Post 1, and Post 2 training conditions. Figure 2c depicts a histogram of button press data across time within the Post 2 condition. Together, this reveals salient time points that subjects perceived as meaningful events in the Post 2 training condition.

To pinpoint the action phases that were most salient across all subjects, each second in the video was categorized by an expert flintknapper (NK) and independently confirmed (DS). This allowed for the temporal categorization of action phases based on one of the six action phases: core reposition, core move, light percussion, percussion, grinding, and tool change. Action phase time points were used to segment the subject x time matrix and categorize them based on the six action phases. The number of subjects that responded with a button press was summed for each specific action phase. The average number of button presses for each action phase was determined across all subjects within a training condition to identify the action phase that subjects’ perceived to be the most meaningful and salient. Using the *shapiro.test* in R, action count violated assumptions of normality (W = 0.60, *p*-value < 0.001). A linear mixed effect model using lmer in R was used to determine statistical significance.

#### Determination of meaningful time points

Due to the hesitation and delayed reaction to an observed evident, the probability of a meaningful event button press was calculated by isolating a 10-s window with a 5-s slide. Using the subject x time matrix, 10 columns (i.e., 10 s) across all subjects were isolated a time, the probability meaningful event button presses were calculated by determining the number of subjects (all the rows) that responded (indicated with a 1) and dividing it by the total number of subjects. The window was shifted by 5-s. The subsequent probability for this 10-s window was calculated using the same metric. The sliding window intervals were applied throughout the entire video resulting in a time series of probability for each training condition (overlaid black lines in Fig. 3a). Time points for analysis were determined by assigning a 90^th^ percentile as the threshold percentile and only used video times points with probabilities at or greater than the 90^th^ percentile (Fig. 3c) indicate probability number value. Together, core move time points that meet the 90^th^ percentile probability threshold or higher were isolated and extracted for continuous gaze analysis (Figure S1). Based on these criteria, we identified 40 core move time points for gaze analysis. Gaze behavior was analyzed for these 40 core move time points in Pre, Post 1, and Post 2 for all subjects for a total of 1,320 data points for each given gaze parameter that was measured.

### Gaze & perceptual analysis

#### Temporal dispersion of gaze using agglomerative hierarchical cluster analysis

We sought to understand the relationship between the dispersion of gaze data across a given core move time point using gaze coefficient of variation and agglomerative hierarchical cluster analysis of gaze position along the x-axis. For the first approach, gaze coefficient of variation was calculated by using continuous gaze data for each of the 40 core move time points and dividing the standard deviation by the mean gaze position across the entire core move continuous data segment. For the second approach, agglomerative hierarchical cluster (AHC) analysis, a bottom-up approach wherein individual data points are clustered based on their similarity, was implemented on continuous x-axis gaze data that has been organized into a time x subject matrix. Data was clustered using XLSTATS 2017.03.44550 with Euclidean distance as the distance measure and Ward’s method as the agglomeration method (Manning et al., 2009;^57^ Tan et al., 2019^58^).

We focused on two classes of parameters: group-level variance and subject-level gaze positions. For the group-level variance, cluster output included within- and between-class variance, which is a percentage value that represents how variable gaze positions are within a cluster and between different clusters respectively. The average within-class and between-class gaze variance across all core move time points that exceeded the probability threshold was used to determine if there was an effect of training on group-level gaze variance. The primary focus of subject-level parameters from the AHC output was central object, which is an actual value for each subject that is closest to the cluster centroid. On average, AHC on continuous core move time points resulted in 3 time clusters with each time cluster containing a time cluster centroid, which is the most statistically representative data point (time point and gaze position) within every identified cluster. Utilization of this approach for all core move time points reduces and summarizes continuous gaze data based on cluster-based descriptive statistics.

#### Spatial dispersion of gaze

To understand where gaze fell in the visual scene during observation, we focused on gaze dispersion. Since core moves are the focal point of subjects’ attention, we set the core itself as the primary area of interest (AOI). More specifically, we deemed the working edge, which is the right most edge of the core, as the subarea of interest because most of the manipulative, tool-use actions applied to mold the core itself occurs within this vicinity. The specific coordinates of the core was determined by an MATLAB2015b-based computer vision object tracking algorithm using machine learning code that utilizes Random Sampling Consensus (RANSAC) for the detection of object features (i.e., core) in a training image and identifying it on a larger visual frame (*Computer Vision Toolbox*^*TM*^ *User’s Guide R2020a*, 2004; “Computer Vision Toolbox - MATLAB & Simulink^29^,” 2020; Bahraini et al., 2018^30^). Video time points that contained core move time points that meet and exceed the 90^th^ percentile probability threshold or higher were cropped and used for frame-by-frame object tracking analysis. A screenshot of the core in various time points within the cropped video were used as training images for the RANSAC machine learning algorithm. Each frame within the cropped video was resized to 1680 × 1050 to match the exact viewing dimensions subjects experienced during video observation. The algorithm extracts features within the training images and applies it to the larger 1680 × 1050 video frame. A box was fitted around the core for each video frame to visually validate proper identification of core coordinates. The x-axis maximum of these coordinates were deemed to the subarea of interest where most of the manipulative, tool-use actions were applied to the core. This procedure was repeated across all 40 core move time points.

These core coordinates were used to calculate the mean gaze distance from the working edge and variance of gaze from the core edge. Subject-level central objects for each identified cluster from the AHC output was used to calculate gaze distance from core coordinate by subtracting the subject-level central object from the working edge coordinates at the corresponding time point of the central object. Gaze variance relative to the core was calculated by calculating the standard deviation of the distance for each time centroid. This procedure was repeated across all 40 core move time points and for each training condition.

### Statistical analysis

Statistical analysis were performed using R version 3.6.2 GUI 1.70 El Capitan build (7735) & RStudio version 1.2.5033. For all values, normality and equality of variances was determined using Shapiro-Wilks and the Levene’s test respectively. Effect sizes for ANOVA was calculated using eta-squared (Ƞ^2^) through the *lsr* R package with type II sum of squares while effect sizes for the Kruskal–Wallis test was calculating using epsilon-squared (**ϵ**). Effect size for pairwise comparisons was calculated using the *effsize* R package by implementing Cohen’s d with Hedge’s g correction, which is utilized for studies with *n* < 20. For statistical test for significance, a *p*-value <0.05 was considered significant for all variables. The data structure and statistical analysis will be discussed in each individual section.

#### Action phase

To determine what action phase subjects deemed meaningful, a linear mixed effect model with maximum likelihood was used to determine the contribution of the fixed effects (action phase and training). A null model, containing only the random effect of Subject, was created using the *lmer* R function to account for any subject-level and baseline differences in behavior. The main effect of action phase and training were examined by creating models for each independent variable, and the interaction effect was determined by the combination of action and training. The chi-square value from the *anova* R function was used to quantify the comparison between the models that incorporated these factors and the baseline model. The *multcomp* and *lsmeans* R packages were used for pairwise comparisons with a Bonferroni adjustment.

#### Temporal dispersion of gaze statistical analysis

Within-class variance is a parameter output from the agglomerative hierarchical clustering that was used to assess gaze dispersion during core moves. Within-class variance was normally distributed (W = 0.99, *p* = 0.26) and exhibited homogeneity of variance (F(2) = 0.72 *p* = 0.49). Dissimilarity, a parameter that is calculated using Euclidean distance and used to quantify gaze distance, was not normally distributed (W = 0.69, *p* < 0.001) but exhibited homogeneity of variance (F(2) = 0.75, *p* = 0.47). Significance was assessed using one-way ANOVA with a TukeyHSD pairwise comparison and a Kruskal-Wallis rank sum test with a Wilcoxon signed ranked post-hoc for within-class variance and dissimilarity respectively.

#### Gaze & motor performance statistical analysis

Each row in the data matrix represents a particular subject, in a specific condition, and during a specific core move time. Due to high variability in the data, a data permutation within a specific condition was conducted. In essence, the subject identifier was shuffled within a given condition. Finally, we took the mean of all dependent variables across all core move time points. Each condition should have a mean coefficient of variation for each subject.

#### Coefficient of variation and mean distance from core

Coefficient of variation (W = 0.94, *p* = 0.084; F(2) = 0.54, *p* = 0.59) and mean distance from the core (W = 0.98, *p* = 0.74; F(2) = 0.23, *p* = 0.80) were both normally distributed and had equal variances. A one-way ANOVA with a TukeyHSD pairwise comparison adjustment was used to determine the effect of training.

#### Gaze variability relative to the core

Gaze variability violated assumptions of normality (W = 0.92, *p* = 0.031) but had equal variances (F(2) = 2.02, *p* = 0.15). Model score also violated assumptions of normality (W = 15.022, *p* < 0.001) but exhibited homogeneity of variance (F(2) = 2.020, *p* = 0.15). For both parameters, a Kruskal-Wallis rank sum test with a Wilcoxon signed ranked post-hoc was conducted to determine significant difference across training conditions.

#### Handaxe score

Model score violated assumptions of normality (W = 0.85, *p* < 0.001) and homogeneity of variance (F(2) = 64.77, *p* < 0.001). A Kruskal-Wallis rank sum test with a Wilcoxon signed ranked post-hoc was conducted to determine significant difference across training conditions.

#### Percussion error

Percussion score violated assumptions of normality (W = 0.90, *p* = 0.008) and exhibited homogeneity of variance (F_2,27_ = 3.17, *p* = 0.06).

### Shift functions

To quantify the extent to which perceptual (i.e., gaze) and motor performance changed from one training condition to another, we utilized shift function originally proposed by Rouselett & colleagues (2017). A shift function is another technique that quantifies how distribution differ by quantifying the amount one distribution must be shifted relative to another distribution (Rousselet et al., 2016, 2017^33,59^). Data are described using deciles wherein each condition is described using 9 quantiles leading to the segmentation of data into 10 different segments. Deciles are calculated using the Harrell-Davis quantile estimator (Wilcox & Erceg-Hurn, 2012). Deciles for one group (e.g., Pre) is compared to a second group (e.g., Post 1) by quantifying the decile differences between the two groups (e.g., Post 1 – Pre). A 95% confidence interval is calculated using percentile bootstrap, and the significance for each decile is quantified using Hochberg’s method (Wilcox & Erceg-Hurn, 2012). In the current study, data was separated into two shifts: Shift1 and Shift2. Shift1 compares gaze and motor performance scores in Pre & Post 1 while Shift2 compares Post 1 and Post 2 data. Each shift data frame was passed through the *rogme* package in R, which implements the Harrell-Davis quantile estimator, 95% confidence interval, and Hochberg’s method.

### Canonical correlation analysis (CCA)

To quantify the strength of the relationships between performance and gaze through training, we conducted canonical correlation analysis (CCA), a multivariate tool that evaluates the relationship between two variables (Wang et al., 2020^60^). Gaze and performance dependent variables were separated into two different variable sets or variates. The performance variate is a subject x performance data matrix with each row representing subjects and the columns representing the three performance variables: learning slope, model score, and percussion score. Similarly, the gaze variate is a subject x gaze matrix with each row representing a subject and each column representing gaze variables: gaze coefficient of variation, mean gaze distance from core, and gaze variability from core. Each training condition (i.e., Pre, Post1, and Post2) consisted of these two performance and gaze matrices yielding a total of six matrices.

For each condition, the performance variate (Xa) and the gaze variate (Xb) were passed through the cca function in MATLAB. Within the cca function, all variables within each variate was z-scored normalized, a common procedure implemented to the data prior to CCA implementation. A sample covariance matrix was created within each variate, which allowed for the implementation of Cholesky factorization using the chol function in MATLAB. Canonical correlations were computed using singular value decomposition through the svd function in MATLAB. The output of the function consisted of canonical weights (Wa, Wb), a numerical value quantifying how a single variable within a variate contributes to the overall trend. The canonical variate (Za, Zb) is a computed weighted sum of original variables obtained by a linear combination of the variables in Xa and another linear combination of variables in Xb. The computed canonical correlation (S), a quantification of the strength of the relationship between the two variates (i.e., performance measures and gaze variables), is obtained by quantifying the Pearson’s R correlation of the canonical variates, Za and Zb. A comparison of weights (Wa, Wb) as well as the changes in correlation between Za and Zb will be assessed from Pre, Post1, and Post2.

### Reporting summary

Further information on research design is available in the Nature Research Reporting Summary linked to this article.

## Supplementary information

Reporting Summary

## Data Availability

The datasets generated during and/or analyzed during the current study are available in Georgia Tech’s SMARTech Respository: (web link: https://smartech.gatech.edu/handle/1853/64500).
