# Supplementary material for: Emergence of perceptuomotor relationships during paleolithic stone toolmaking learning: intersections of observation and practice
Source: Commun Biol. 2021 Nov 11;4:1278. doi: 10.1038/s42003-021-02768-w (PMC8585878; doi:10.1038/s42003-021-02768-w)
Supplement: Supplementary file 1 — Reporting Summary [file 42003_2021_2768_MOESM1_ESM.pdf]

nature research

Corresponding author(s): Lewis A Wheaton

Last updated by author(s): Sep 30, 2021

Reporting Summary

Nature Research wishes to improve the reproducibility of the work that we publish. This form provides structure for consistency and transparency in reporting. For further information on Nature Research policies, see our Editorial Policies and the Editorial Policy Checklist.

Statistics

For all statistical analyses, confirm that the following items are present in the figure legend, table legend, main text, or Methods section.

n/a	Confirmed	
☐	☒	The exact sample size (*n*) for each experimental group/condition, given as a discrete number and unit of measurement
☐	☒	A statement on whether measurements were taken from distinct samples or whether the same sample was measured repeatedly
☐	☒	The statistical test(s) used AND whether they are one- or two-sided<br>*Only common tests should be described solely by name; describe more complex techniques in the Methods section.*
☐	☒	A description of all covariates tested
☐	☒	A description of any assumptions or corrections, such as tests of normality and adjustment for multiple comparisons
☐	☒	A full description of the statistical parameters including central tendency (e.g. means) or other basic estimates (e.g. regression coefficient) AND variation (e.g. standard deviation) or associated estimates of uncertainty (e.g. confidence intervals)
☐	☒	For null hypothesis testing, the test statistic (e.g. *F*, *t*, *r*) with confidence intervals, effect sizes, degrees of freedom and *P* value noted<br>*Give P values as exact values whenever suitable.*
☐	☒	For Bayesian analysis, information on the choice of priors and Markov chain Monte Carlo settings
☐	☒	For hierarchical and complex designs, identification of the appropriate level for tests and full reporting of outcomes
☐	☒	Estimates of effect sizes (e.g. Cohen's *d*, Pearson's *r*), indicating how they were calculated

Our web collection on statistics for biologists contains articles on many of the points above.

Software and code

Policy information about availability of computer code

Data collection	Data collection was done through Gazepoint software
Data analysis	MATLAB was used for all analysis, with R for statistical reporting.

For manuscripts utilizing custom algorithms or software that are central to the research but not yet described in published literature, software must be made available to editors and reviewers. We strongly encourage code deposition in a community repository (e.g. GitHub). See the Nature Research guidelines for submitting code & software for further information.

Data

Policy information about availability of data

All manuscripts must include a data availability statement. This statement should provide the following information, where applicable:

- Accession codes, unique identifiers, or web links for publicly available datasets
- A list of figures that have associated raw data
- A description of any restrictions on data availability

Datasets generated during the current study are available in the Georgia Tech SMARTech repository at https://www.dropbox.com/sh/hk74t2nhpd9ss7y/AADJzUKGDGwXjuLMfsSFIt8pa?dl=0

Field-specific reporting

Behavioural & social sciences study design

All studies must disclose on these points even when the disclosure is negative.

Study description	This is a qualitative, longitudinal study
Research sample	The research sample was drawn from the Emory University faculty and staff, and those in the surrounding community. Participants were 10 female/7 male; 21-48 years of age, median 27.25 years. All participants were right-handed, had no prior knapping experience, and provided written informed consent.
Sampling strategy	This represents a convenience sample. These samples are based on similar work done by the labs of the co-authors that suggest the ability to detect patterns of motor learning in ~10 participants. Given the long degree of training (100 hours) we recruited 17 participants knowing that some would drop out due to the demands of the task.
Data collection	While watching the videos, during Pre, Post 1 and Post 2, participants indicated their perception of meaningful actions using the left and right arrows on the computer keyboard. Keyboard button presses for meaningful events were used to pinpoint the action phases and time points that were deemed most salient to subjects. Button press latencies are time points that correspond to specific time points in the video and were collected from the start until the end of the video. Latencies were stored matrix wherein columns represented each subject and each row corresponds to a specific time the subject responded to a meaningful event. The Gazepoint control system (GP3), a 60-Hz high-performance eye tracking platform, was placed below the computer monitor approximately 65cm or an arms distance away from the subject. The eye tracker was stabilized with a mini tripod that allowed researchers to adjust the position of the eye tracker based on each individual subjects' height. A laptop was used to power and obtain data from the eye tracker through two USB cables. An experimenter was present for all data collection, and sat silently to ensure data collection continued adequately
Timing	Data collection began around 12/2015 and continued through around 8/2016
Data exclusions	Participants were excluded based on whether they were able to complete training.
Non-participation	6 participants left the study because of difficulties scheduling training dates. There were no adverse events.
Randomization	Participants are not allocated into experimental groups.

Reporting for specific materials, systems and methods

We require information from authors about some types of materials, experimental systems and methods used in many studies. Here, indicate whether each material, system or method listed is relevant to your study. If you are not sure if a list item applies to your research, read the appropriate section before selecting a response.

Materials & experimental systems

n/a	Involved in the study
☒	☐ Antibodies
☒	☐ Eukaryotic cell lines
☒	☐ Palaeontology and archaeology
☒	☐ Animals and other organisms
☐	☒ Human research participants
☒	☐ Clinical data
☒	☐ Dual use research of concern

Methods

n/a	Involved in the study
☒	☐ ChIP-seq
☒	☐ Flow cytometry
☒	☐ MRI-based neuroimaging

Human research participants

Policy information about studies involving human research participants

Population characteristics	See above
Recruitment	Participants were recruited by word of mouth and fliers on the Emory University Campus.
Ethics oversight	The Emory University IRB approved the study, which reciprocated to the Georgia Tech IRB.

Note that full information on the approval of the study protocol must also be provided in the manuscript.
